# Mesalazine-induced myocarditis in a patient with ulcerative colitis: a case report

**DOI:** 10.1093/ehjcr/ytae458

**Published:** 2024-08-27

**Authors:** Simon Littlewood, Evgenia Nikolou, Waqar Aziz, Lisa Anderson

**Affiliations:** School of Biomedical Engineering and Image Science, King's College London, 3rd Floor Lambeth Wing, St Thomas’ Hospital, London SE1 7EH, United Kingdom; Department of Cardiology, St George's Hospital, Blackshaw Road, London SW17 0QT, United Kingdom; Department of Cardiology, St George's Hospital, Blackshaw Road, London SW17 0QT, United Kingdom; Department of Cardiology, St George's Hospital, Blackshaw Road, London SW17 0QT, United Kingdom; Department of Cardiology, St George's Hospital, Blackshaw Road, London SW17 0QT, United Kingdom

**Keywords:** Case report, Myocarditis, Mesalazine, Inflammatory bowel disease, Imaging, Cardiac MRI

## Abstract

**Background:**

Mesalazine is an established first-line therapy for inflammatory bowel disease (IBD) and remains the mainstay of treatment for mild to moderate ulcerative colitis (UC). Both mesalazine and UC are rare but recognized causes of myopericarditis. Cardiac magnetic resonance (CMR) is a non-invasive method of assessing for myopericarditis. This case reports highlights the importance of early CMR in diagnosis, and management of myocarditis in a patient with IBD.

**Case summary:**

A 28-year-old male was admitted with a 2-day history of chest pain. Three weeks prior to this presentation, the patient was initiated on mesalazine for UC. Serum troponin T and C-reactive protein were elevated. An echocardiogram showed borderline low left ventricular systolic function (LVEF = 50–55%). A CMR showed extensive patchy late gadolinium enhancement (LGE) in the mid to epicardial basal and mid lateral wall. The findings were consistent with acute myocarditis, and a working diagnosis of mesalazine-induced myocarditis was made. Mesalazine was stopped and guideline-directed anti-inflammatories initiated. Oral prednisolone was also introduced for IBD control. Follow-up CMR at four months showed near complete resolution of LGE.

**Discussion:**

Myocarditis in the context of IBD may be infective, immune-mediated or due to mesalazine hypersensitivity. Histological conformation was not available in this case. This case report highlights the importance of access to early CMR in order establish the diagnosis and withdrawal of the culprit medication. In the majority of cases, CMR will replace the need for endomyocardial biopsy; however, this may still be required in the most severe cases.

**Learning points**

- Mesalazine is a rare but recognized cause of myocarditis.
- Cardiac magnetic resonance (CMR) imaging non-invasive investigation of choice for making the diagnosis of myocarditis.
- Early CMR helps avoid other unnecessary and potentially invasive investigations.
- Treatment involves withdrawal of mesalazine and initiation of anti-inflammatory medications.
- Liaison with specialists in gastroenterology is essential to avoid inflammatory bowel disease flare.

## Introduction

Mesalazine, also known as mesalamine and 5-aminosalicylic-acid (5-ASA), is an established first-line therapy for inflammatory bowel disease (IBD) and remains the mainstay of treatment for mild to moderate ulcerative colitis (UC).^1^ Although the exact mechanism of action is not fully understood, it is thought to act by inhibiting the synthesis of prostaglandins and leukotrienes thereby modulating the inflammatory response associated with the cyclooxygenase and lipoxygenase pathways in the colonic mucosa.^1^ Mesalazine is useful for controlling active inflammation and maintaining remission and has the advantage of being generally well-tolerated and safe for long-term use.^1^

Major adverse side effects are rare but can include interstitial nephritis, pancreatitis, hepatitis, and blood dyscrasias.^2^ Cardiac inflammation is also a very rare side effect of mesalazine use, with an incidence up to 0.3% previously reported, but with potentially fatal consequences.^3,4^ Ulcerative colitis itself is associated with extra-intestinal manifestations of which myocarditis is a very rare recognized association with an incidence of 0.04% providing a diagnostic challenge for the treating physician.^5,6^

Imaging with cardiac magnetic resonance (CMR) offers a non-invasive approach and can confirm characteristic imaging features in the setting of myocardial inflammation.^7^ We present a case of acute myocarditis following the initiation of mesalazine therapy. Although similar cases exist, this one is notable for its distinctive clinical presentation and the crucial role of early CMR imaging in diagnosis and management. This underscores CMR’s importance in detecting rare drug-induced cardiac complications and provides valuable insights for improving clinical awareness and management.

## Summary figure

**Figure:**
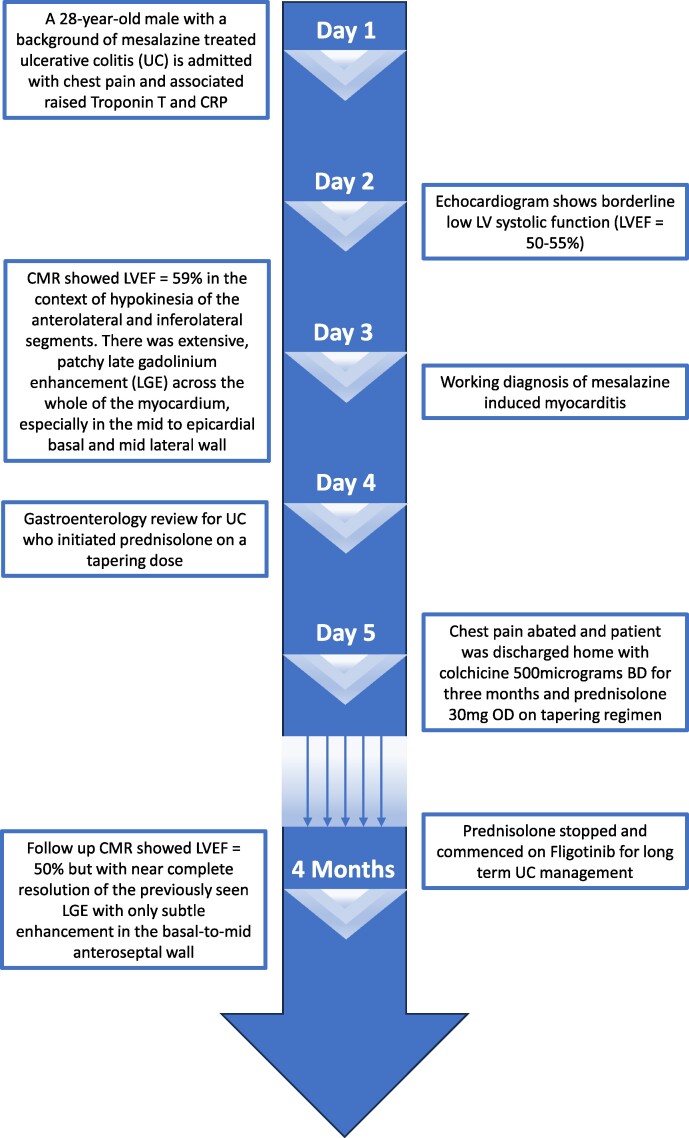


## Case presentation

A 28-year-old male was admitted following a 2-day history of intermittent sharp, central chest pain with associated diaphoresis. He attended the Emergency Department via ambulance. On further questioning, the pain was pleuritic in character and was exacerbated by sitting forwards. He reported no viral symptoms during the preceding three months and had not received any COVID-19 booster vaccinations. His past medical history was notable for a new diagnosis of UC, for which he had been started on mesalazine three weeks prior to this presentation resulting in an improvement in his bowel symptoms. He took no other regular medication and reported no allergies. He was a smoker of 3–4 cigarettes a day, drank no alcohol nor took any recreational drugs. There was no family history of inherited or acquired cardiovascular disease.

The patient was haemodynamically stable, and detailed clinical examination was unremarkable other than Stage 2 finger clubbing.

**Figure 1:**
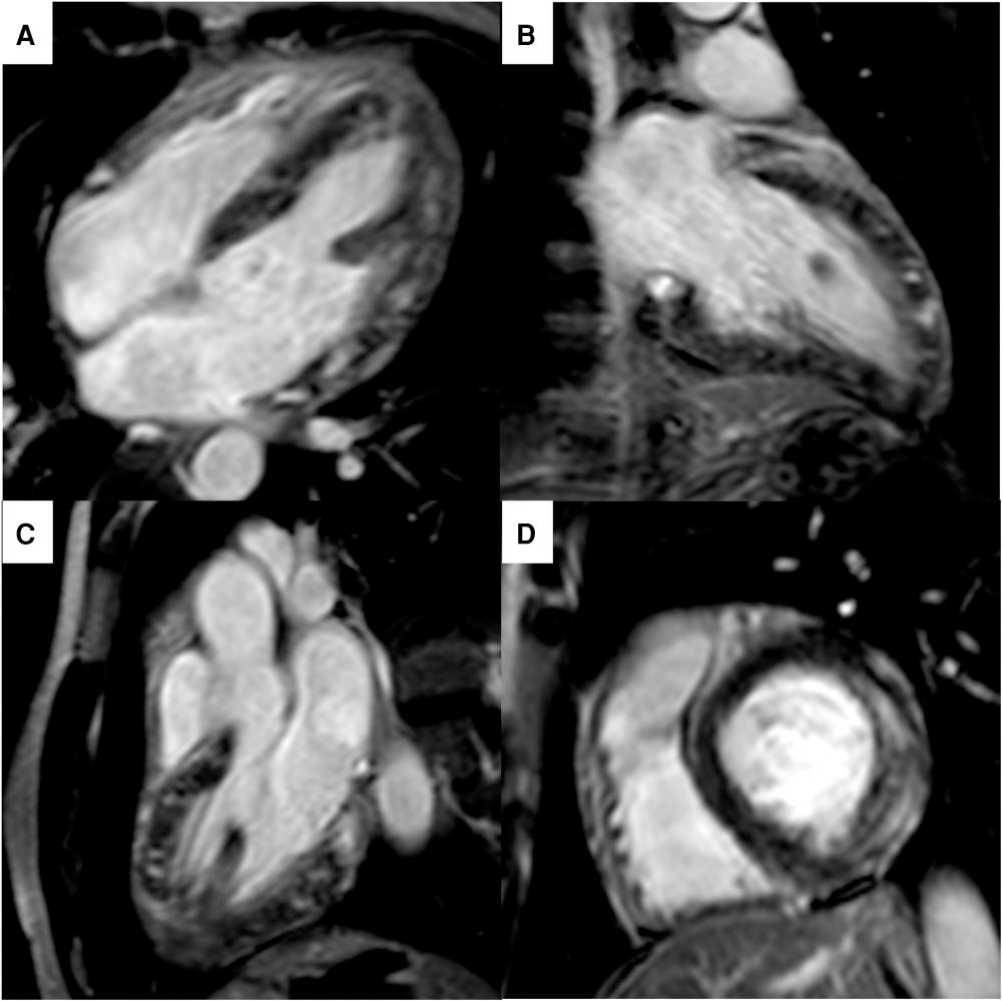
Late gadolinium enhanced cardiac MRI images acquired 3 days following admission. Four-chamber (*A*), three-chamber (*B*), two-chamber (*C*), and basal short axis (*D*) views showing extensive, patchy enhancement in the mid-wall and sub-epicardium. Short axis slice in the basal ventricle (*D*) shows prominent mid-wall and sub-epicardial enhancement in the lateral wall. The lateral wall is visibly thickened compared to the septum (*D*).

ECG showed sinus rhythm with benign early repolarization in the anterior leads. Chest X-ray was unremarkable. Blood tests showed Hb of 132 g/L (120–170 g/L), low mean corpuscular volume of 75 fL (80–97 fL), and raised high-sensitivity cardiac troponin T concentration of 275 ng/L (<14 ng/L), which peaked at 632 ng/L on Day 2 of admission. C-reactive protein was raised at 59 mg/L (<5 mg/L) and peaked at 173 mg/L also on Day 2 of admission. Serum viral and auto-antibody screens were negative. An echocardiogram showed normal left ventricular (LV) size and wall thickness with borderline low systolic function (LVEF = 50–55%) in the context of dyssynchronous septal wall motion. The right ventricle (RV) and the atria were of normal size and function. Valvular assessment was also normal, and there was no evidence of pericardial effusion. Considering the history of chest pain, repolarization changes on ECG, and troponin rise, we considered performing an invasive coronary angiogram to investigate a possible non-ST elevation myocardial infarction. However, due to the atypical nature of the chest pain and young age of the patient, this was felt to be a less likely differential diagnosis leading to the decision to proceed with a CMR instead. On Day 3 of admission, a CMR was performed that showed an LV of normal size with mild systolic dysfunction and normal ejection fraction (LVEF = 59%) in the context of hypokinesia of the anterolateral and inferolateral segments (see Supplementary material online, *Videos S3A* and *S3B*). There was increased wall thickness of 14 mm in the basal lateral wall, and the RV was small with normal systolic function. There was extensive, patchy late gadolinium enhancement (LGE) across the whole of the myocardium, especially in the mid to epicardial basal and mid lateral wall (*Figure 1*). T2-weighted STIR imaging for oedema was suboptimal; however, appeared to demonstrate increased signal intensity in the anterior wall. There was no pericardial enhancement or pericardial effusion. The findings were consistent with an extensive inflammatory myocardial process. Endomyocardial biopsy was considered but not performed due to the patient’s haemodynamic stability and convincing CMR findings.

A working diagnosis of mesalazine-induced myocarditis was made. Mesalazine was stopped and the patient was commenced on ibuprofen 600 mg three times daily, which was ceased after resolution of pain on Day 3 of hospital admission, and a course of colchicine 500 μg twice daily was prescribed for three months with proton-pump inhibitor gastroprotection. As LVEF was preserved and there were no arrhythmias, cardio-protective medications were not initiated. He was reviewed by the gastroenterology team on Day 4, who initiated prednisolone 30 mg once daily on a tapering dose regimen to manage his IBD. The patient was advised to refrain from strenuous exercise for six months. His symptoms improved, and he was successfully discharged from the hospital on Day 5.

**Figure 2:**
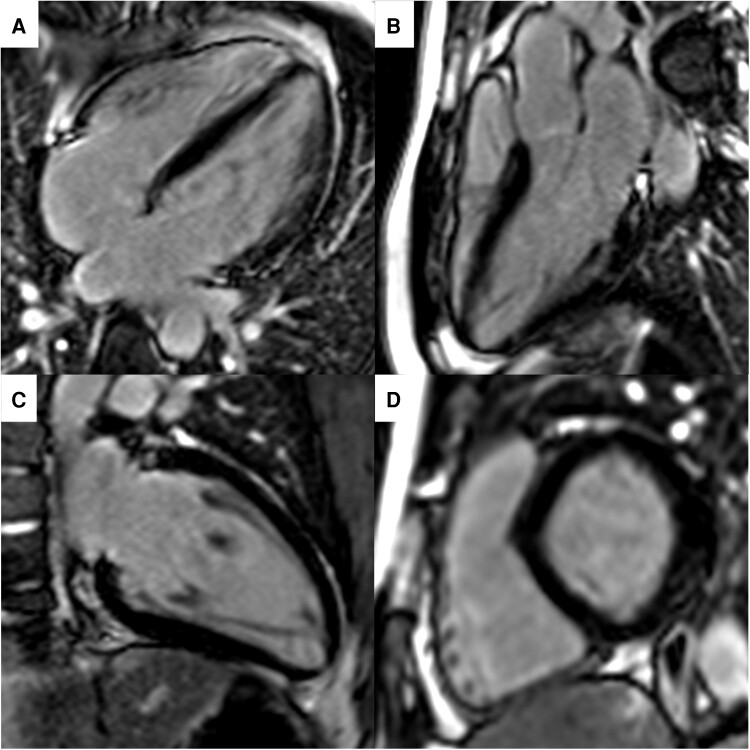
Late gadolinium enhanced cardiac MRI images acquired four months following discharge from hospital. Four-chamber (*A*), three-chamber (*B*), two-chamber (*C*), and basal short axis (*D*) views show subtle patchy enhancement in the basal-to-mid lateral wall. Short axis slice in the basal ventricle (h) also demonstrates subtle enhancement in the basal lateral wall. The lateral wall is now of normal thickness.

At four-month follow-up, he was well and had returned to work, but continued to report occasional mild chest discomfort. Repeat imaging with CMR at four months showed normal LV size with mildly impaired global systolic function (LVEF = 50%) and normal RV size with preserved systolic function (see Supplementary material online, *Videos S4A* and *S4B*). The previously thickened lateral wall had normalized (6 mm), and the extensive LGE had almost completely resolved with only subtle enhancement in the basal-to-mid anteroseptal wall (*Figure 2*). These findings were consistent with the resolution of myocardial inflammation and only mild residual patchy fibrosis of the basal-to-mid anteroseptal wall. He was started on filgotinib (Janus-associated tyrosine kinase inhibitor) by the gastroenterology team for ongoing management of UC.

## Discussion

The aetiology of myocarditis can be challenging for clinicians, and often no clear cause is found. In this case, the underlying IBD or mesalazine use is likely culprits but mesalazine as the underlying aetiology is made more likely due to the initiation three weeks prior to this presentation, which is in keeping with the timeline of between two and four weeks from medication initiation to symptom onset in previously published reports.^8–11^ Myocarditis as an extra-intestinal manifestation of UC is also less likely due to the patient’s improved bowel symptoms at the time of presentation rather than active disease flare as described in previous case reports.^12–15^ The proposed mechanism of mesalazine-induced cardiac inflammation is thought to be a cell-mediated hypersensitivity reaction rather than direct cardiac toxicity, and this is supported by the findings of eosinophilic infiltration on endomyocardial biopsy.^16^ Cardiac magnetic resonance was essential in this case for the diagnosis of myocarditis. Endomyocardial biopsy remains the gold standard investigation however, using the updated 2018 Lake Louise Criteria (LLC)^7^ with a sensitivity of 87.5% and specificity of 96.2%, this can now be avoided in the majority of cases.^17^ The Modified LLC requires demonstration of (i) myocardial oedema using T2-mapping or T2-weighted imaging and (ii) markers of non-ischaemic myocardial injury on T1-weighted imaging, such as elevated native T1 times of extracellular volume on T1-mapping or non-ischaemic pattern of LGE. Other supportive criteria include evidence of pericarditis determined either by the presence of effusion or by an abnormal appearance on LGE, T2 or T1 sequences alongside regional or global wall motion abnormalities. In our case, although T2-weighted STIR images were of suboptimal quality, they were strongly suggestive of myocardial oedema which along with a non-ischaemic pattern of LGE and regional wall motion abnormalities, met the LLC criteria for myocarditis. Suboptimal T2-weighted STIR imaging is a common issue, particularly when using 3 T scanners as in this case, due to increased radiofrequency (RF) field inhomogeneity resulting in signal variation across the myocardium. Similarly, the increased magnetic field (B_0_) inhomogeneity may result in inhomogeneous fat suppression across the area of interest.^18^ Parametric T2-mapping is a more robust technique for oedema assessment^19^ that unfortunately was not acquired in our case. T2-mapping and T1-weighted imaging are powerful tools in differentiating myocarditis from other cardiac conditions. T2-mapping is particularly useful for identifying acute inflammation/oedema, whereas T1-mapping is sensitive to both acute and chronic tissue changes. However, these techniques have several limitations including overlap with other conditions, such as acute myocardial infarction (MI) and infiltrative diseases. These techniques when used in combination, alongside LGE imaging to determine scar pattern, can help distinguish myocarditis from other conditions. In this case, using CMR to exclude MI also helped the patient avoid an invasive coronary angiogram. Cardiac inflammatory syndromes often evolve over days to weeks followed by resolution but may also lead to acute severe left ventricular dysfunction or transition to chronic dilated cardiomyopathy.^20^ Therefore, the optimal sensitivity of diagnostic CMR imaging is up to two weeks from presentation, although if possible, this should be completed during the index admission. There is also useful prognostic information to be gained in this early window with the presence of myocardial oedema without LGE associated with improved recovery and outcomes.^21^ Early use of CMR also helped inform treatment decisions, by ruling out other potential differentials for chest pain and serum troponin rise such as MI or infiltrative disease. In this case, CMR led to the withdrawal of mesalazine along with initiation of treatment with ibuprofen and colchicine, as per European Society of Cardiology (ESC) guidelines for the management of myocarditis,^22^ leading to rapid resolution of symptoms that correlated with improvement in cardiac biomarkers. Oral steroids were also introduced as part of UC management following the acute withdrawal of mesalazine as a bridge to second-line therapy, although it is likely that these also contributed to the resolution of cardiac symptoms as they are recommended as second-line therapy for myocarditis in ESC guidelines.^22^ There are no specific guidelines on whether re-challenging with mesalazine is advised and this should be approached with caution due to the risk of recurrence that can occur within hours or days.^23^

## Conclusion

Mesalazine is a rare but recognized cause of myocarditis. Withdrawal of medication leads to a rapid resolution of symptoms. This clinical report highlights the importance of early CMR to confirm the diagnosis and helps rule out other potentially serious diagnoses, allowing for rapid withdrawal of the culprit agent and potential avoidance of unnecessary invasive investigations. Liaison with colleagues in gastroenterology is crucial for avoidance of IBD disease flare and ongoing management with second-line agents. This report emphasizes the need for greater access to CMR in the acute phase of illness to streamline management and future investigation. The establishment of an international registry of patients with drug-induced myocarditis may help to facilitate larger-scale studies to establish potential biomarkers of susceptibility, treatment guidelines, and long-term outcome data.

## Supplementary Material

ytae458_Supplementary_Data

## Data Availability

The data underlying this article are available in the article and in the online Supplementary material.

## Lead author biography

I am a Specialist Registrar in Cardiology and Clinical Research Fellow in the UK. My interests include novel cardiac MRI techniques for the detection of coronary artery disease and advanced imaging for coronary intervention.

## Supplementary material

**Consent:** The patient provided written informed consent for the anonymous use of images for the purpose of publication and educational purposes in accordance with COPE guidelines.

**Funding:** The authors declare no funding or conflicts of interest for this case report.
